# NMR Spectroscopy for Protein Higher Order Structure Similarity Assessment in Formulated Drug Products

**DOI:** 10.3390/molecules26144251

**Published:** 2021-07-13

**Authors:** Deyun Wang, You Zhuo, Mike Karfunkle, Sharadrao M. Patil, Cameron J. Smith, David A. Keire, Kang Chen

**Affiliations:** 1 Northeast Medical Products Laboratory, Office of Regulatory Science, Office of Regulatory Affairs, U.S. Food and Drug Administration, Jamaica, NY 11433, USA; deyun.wang@fda.hhs.gov; 2 Division of Complex Drug Analysis, Office of Testing and Research, Office of Pharmaceutical Quality, Center for Drug Evaluation and Research, U.S. Food and Drug Administration, Silver Spring, MD 20993, USA; you.zhuo@fda.hhs.gov (Y.Z.); sharadmpatil@gmail.com (S.M.P.); 3 Division of Pharmaceutical Analysis, Office of Testing and Research, Office of Pharmaceutical Quality, Center for Drug Evaluation and Research, U.S. Food and Drug Administration, St. Louis, MO 63110, USA; mike.karfunkle@fda.hhs.gov; 4 Division of Liquid Based Products I, Office of Lifecycle Drug Products, Office of Pharmaceutical Quality, Center for Drug Evaluation and Research, U.S. Food and Drug Administration, Silver Spring, MD 20993, USA; cameron.smith@fda.hhs.gov; 5 Office of Testing and Research, Office of Pharmaceutical Quality, Center for Drug Evaluation and Research, U.S. Food and Drug Administration, St. Louis, MO 63110, USA; david.keire@fda.hhs.gov

**Keywords:** similarity metrics, Mahalanobis distance, chemical shift difference, peak profile, relative peak height

## Abstract

Peptide and protein drug molecules fold into higher order structures (HOS) in formulation and these folded structures are often critical for drug efficacy and safety. Generic or biosimilar drug products (DPs) need to show similar HOS to the reference product. The solution NMR spectroscopy is a non-invasive, chemically and structurally specific analytical method that is ideal for characterizing protein therapeutics in formulation. However, only limited NMR studies have been performed directly on marketed DPs and questions remain on how to quantitively define similarity. Here, NMR spectra were collected on marketed peptide and protein DPs, including calcitonin-salmon, liraglutide, teriparatide, exenatide, insulin glargine and rituximab. The 1D ^1^H spectral pattern readily revealed protein HOS heterogeneity, exchange and oligomerization in the different formulations. Principal component analysis (PCA) applied to two rituximab DPs showed consistent results with the previously demonstrated similarity metrics of Mahalanobis distance (D_M_) of 3.3. The 2D ^1^H-^13^C HSQC spectral comparison of insulin glargine DPs provided similarity metrics for chemical shift difference (Δδ) and methyl peak profile, i.e., 4 ppb for ^1^H, 15 ppb for ^13^C and 98% peaks with equivalent peak height. Finally, 2D ^1^H-^15^N sofast HMQC was demonstrated as a sensitive method for comparison of small protein HOS. The application of NMR procedures and chemometric analysis on therapeutic proteins offer quantitative similarity assessments of DPs with practically achievable similarity metrics.

## 1. Introduction

Complex generic and biosimilar drug products (DPs) are increasingly developed and comprehensive analysis of these DPs is the foundation for their regulatory approval [1,2,3,4]. The active pharmaceutical ingredient (API) or drug substance (DS) in protein DPs ranges in size from short peptides to large monoclonal antibodies (mAbs). The native folding of proteins, heterogeneity, dynamic exchange between conformations, oligomerization and aggregation profile in a formulation are collectively called the higher order structure (HOS) properties of protein therapeutics and are typically critical for efficacy and safety [5].

Protein HOS is stabilized by weak hydrogen bonding, electrostatic and hydrophobic forces, which are solvent dependent, and, consequently, formulation differences affect HOS [6,7,8,9]. In addition, proteins can be chemically modified either purposely, e.g., pegylation, or unintentionally, e.g., oxidation, which could introduce variability to protein HOS [10,11,12]. All these factors and the accompanying sensitivity to solution conditions necessitate characterizing the protein chemistry and HOS with minimal perturbation to the formulation by ideally using DPs [13]. In addition, the analytical means to assess protein HOS in a formulation are desired for generic and biosimilar drug developers that mostly only have access to the marketed originator DPs that are usually deemed as the reference DPs. 

With the development of higher field strength magnets and cryogenic probes, modern high-resolution NMR spectroscopy is a non-invasive and sensitive method for protein molecular structure characterization [14,15,16,17,18]. However, several assumptions among stakeholders have limited the application of NMR on formulated DPs. The first is that strong excipient signals in a DP would interfere with weak DS signals such that NMR spectra would be dominated by the peaks of the excipients and would not be useful for protein HOS assessment. Indeed, NMR for protein HOS characterizations [19] has been applied on proteins extracted from DP [20,21,22], proprietary DS or non-marketed DP [23,24,25], which aimed to demonstrate the applicability of modern heteronuclear NMR to characterize proteins with ^15^N and ^13^C nuclei at natural abundances. 

Second, the lack of acceptable metrics for similarity assessment means that most comparisons have been made at a visual level. The question of the level of similarity that is practically measurable remains to be answered quantitively. Previous attempts were made to collect NMR spectra on a DS enriched formulation of filgrastim [26] and DP formulations of insulin [27]. A combined chemical shift difference of 8 ppb or less was proposed as the threshold for experimental precision in 2D-NMR comparisons of biosimilars using data between the US and Indian marketed filgrastim DPs [26]. The principal component analysis (PCA) of the insulin DP NMR spectra revealed the practically achievable similarity threshold expressed in Mahalanobis distance (D_M_) to be 3.3 or less [27]. These values were achieved when 600 MHz spectrometer with room temperature probe was used, therefore, the derived metrics were practical (in terms of the availability of instruments) and could be useful in establishing the acceptance criteria for a certain DP before and after a manufacturing change and for the comparison between a generic or biosimilar protein and the reference DP. However, their validity has not been further tested. 

Third, the type of HOS properties reliably measured from DPs using modern NMR is not entirely clear. Herein, 1D ^1^H NMR spectra were acquired on a range of marketed DPs with protein molecular weights ranging from 3 kDa to 145 kDa and with the protein concentration as low as 0.01 mM. The protein HOS properties of folding, intermediate exchange and oligomerization were all reflected in the NMR spectral patterns. Using rituximab DPs, the proposed D_M_ similarity metric was verified again. Using insulin glargine DPs, the methyl peak profile method showed that both chemical shift and relative peak height can be used to derive practically achievable similarity metrics. Finally, the sensitive ^1^H-^15^N sofast HMQC experiment was demonstrated to be a valuable NMR method to characterize the protein backbone HOS.

## 2. Results

The peptide and protein drug products (DPs) listed in Table 1 were sourced from the US market except Reditux^®^, which was sourced from India. All DPs are the reference drugs except Basaglar^®^ and Reditux^®^, which are follow-on products to Lantus^®^ and Rituxan^®^, respectively. All 1D ^1^H, 2D ^1^H-^13^C and 2D ^1^H-^15^N NMR spectra were collected on formulated DPs with minimal dilution of adding 5% D_2_O (*v*/*v*).

### 2.1. 1D ^1^H NMR Spectroscopy

#### 2.1.1. Excipients

**Figure 1:**
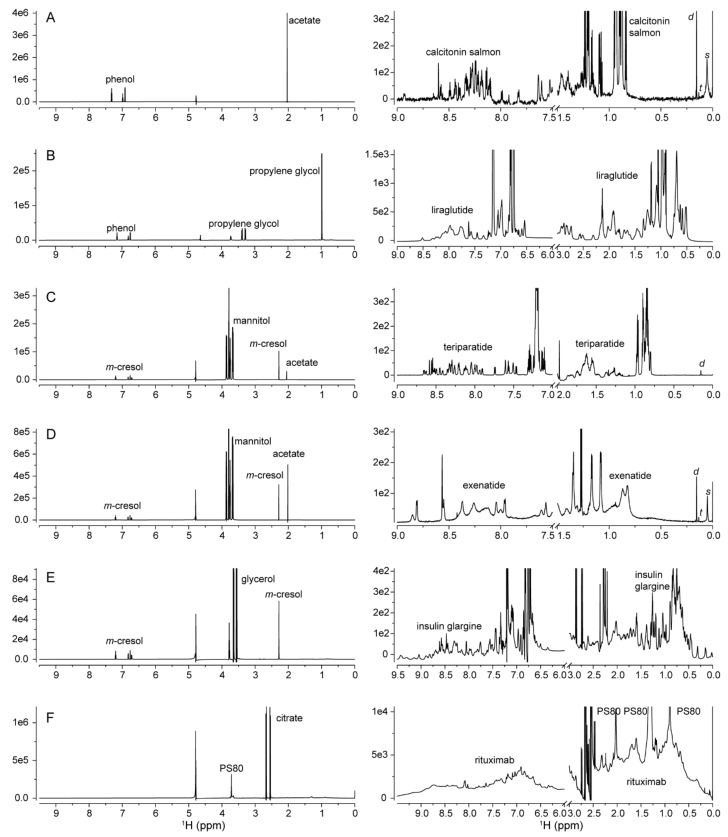
The 1D ^1^H NMR spectra of protein drug products of Miacalcin^®^ (**A**), Saxenda^®^ (**B**), Forteo^®^ (**C**), Byetta^®^ (**D**), Lantus^®^ (**E**) and Rituxan^®^ (**F**) collected using an 850 MHz spectrometer. The spectra on the left are in full scale and those on the right are vertically enlarged and horizontally cut to display protein peaks. Signals from major excipients, drug substances and the leachable compounds dimethylsilanediol (d), trimethylsilanol (t) and polydimethylsiloxane (s) are annotated.

Excipients in protein formulations can function as preservatives (e.g., phenol and *m*-cresol), tonicity agents (e.g., mannitol), pH buffering agents (e.g., acetate) or protein stabilizers (e.g., polysorbate 80) [28]. The excipients are mostly small molecules at high concentration relative to the API. Due to the fast tumbling of excipients in liquid formulations, excipient peaks generally were sharper and more intense in the NMR spectra (Figure 1, left panels). Most excipient peaks were located in the high field region between 1 and 4 ppm, while preservatives with aromatic moieties had peaks between 6.5 and 7.5 ppm. The peaks were readily assignable with the help of chemical shift databases [29,30] or 2D ^1^H-^13^C spectra. The excipient polysorbate 80 (PS80) had a more complicated spectrum, with major peaks at 3.7, 2.3, 2.0, 1.6, 1.3 and 0.9 ppm [31]. Importantly, all excipient peaks should be excluded when protein HOS comparison is performed.

#### 2.1.2. Process-Related Impurities

Small sharp peaks from process related impurities such as residual solvents and leachable were also identified and should be blinded out of HOS comparison as well [32,33]. For example, silicone oil used as a lubricant in DP containers could leach into the formulation and appear as a broad peak of polydimethylsiloxane (s) at 0.05 ppm, which can be further hydrolyzed to dimethylsilanediol and trimethylsilanol and appears as sharp peaks at 0.15 ppm (d) and 0.13 ppm (t), respectively (Figure 1A,C,D, right panels) [34]. Notably, the proton resonances of larger proteins could overlap with the spectral region around 0 ppm. Therefore, if NMR is used for the quantification of silicone oil components in protein formulations, T_2_-filtered CPMG pulse train may be used to remove protein resonances [35,36].

#### 2.1.3. Protein HOS

The protein DS may be formulated at concentration of about 1 mM or less (Table 1). The 1D ^1^H NMR spectrum is the most sensitive NMR method to characterize protein HOS in DP formulations. The spectra need to be vertically enlarged by 2–4 orders of magnitude in order to visualize the lower intensity protein peaks (Figure 1, right panels). Among the tested DPs, the 3.43 kDa calcitonin-salmon is formulated at the lowest concentration of 9.7 μM. Calcitonin’s sharp and dispersed amide peaks suggested that calcitonin-salmon adopts a folded monomeric HOS in formulation (Figure 1A). The 3.75 kDa liraglutide has a similar M.W. to calcitonin-salmon, however, broadened amide peaks were observed in liraglutide’s spectra (Figure 1B), suggesting oligomerization of the protein in formulation. 

The 4.12 kDa teriparatide had sharp and dispersed amide peaks, suggesting a folded HOS in the formulation (Figure 1C). For the 4.19 kDa exenatide, much broadened peaks were observed while the detected number of peaks was much less (Figure 1D), suggesting the peptide was undergoing intermediate exchange broadening [37]. The observed exchange broadening is associated with exenatide in equilibrium between several HOS states and the exchange kinetics occur over a similar time scale of the chemical shift difference between different states, usually in the range of μs-ms exchange. 

For the 6.06 kDa insulin glargine, the detected dispersed peaks suggest well folded HOS in the formulation at pH 4 (Figure 1E). Finally, the observed broadened peaks of the 145 kDa rituximab were due to its large M.W., but the dispersed amide peaks suggest the monoclonal antibody has a folded HOS (Figure 1F).

#### 2.1.4. Similarity Metrics of D_M_

Although the 1D ^1^H spectra can be used to assess protein HOS qualitatively, a quantifiable similarity metric is of interest to demonstrate comparability after manufacturing changes or similarity between any two drug brands [38]. Previously, 1D ^1^H spectra between the reference insulin and the follow-on insulin DPs were chemometrically compared using principal component analysis (PCA) and Mahalanobis distance (D_M_) metrics, which were derived from PCA space [27]. The previous results on insulin DPs suggested a D_M_ value of 3.3 as the similarity threshold [27], where above 3.3 value there were clear differences in the HOS, while below that there were no discernable differences. Here, the rituximab DPs Rituxan^®^ and Reditux^®^ were compared using the same approach. Excipient free regions of the spectra including the down field amide spectrum are informative for rituximab HOS.

**Figure 2:**
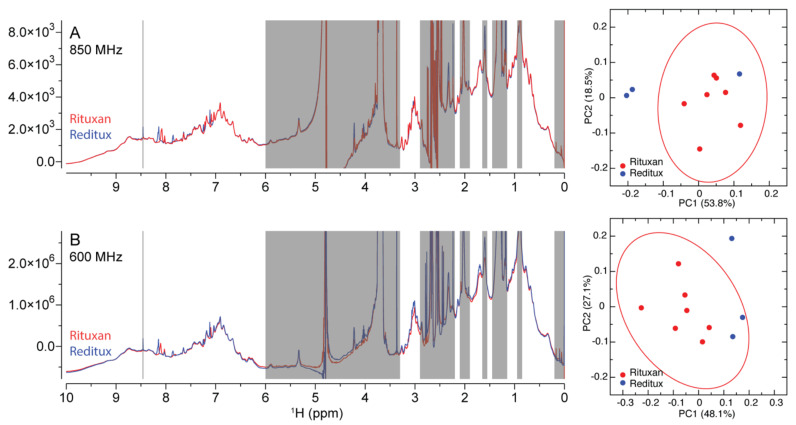
The superimposed 1D ^1^H NMR spectra of representative rituximab drug products (DP) of Rituxan^®^ and Reditux^®^ collected using 850 MHz (**A**, left) and 600 MHz (**B**, left) spectrometers. The spectral regions in gray of 0–0.2 ppm, 0.85–0.95 ppm, 1.15–1.45 ppm, 1.55–1.65 ppm, 1.9–2.1 ppm, 2.2–2.9 ppm, 3.3–6 ppm and 8.45–8.47 ppm were excluded before principal component analysis (PCA). The resulting PCA scores for each DP lot from both 850 MHz data (**A**, right) and 600 MHz data (**B**, right) were plotted along the PC1 and PC2 axes. The 90% confidence ellipses are drawn for Rituxan^®^ lots only (A/B, right).

The 1D ^1^H spectra of seven lots of Rituxan^®^ and three lots of Reditux^®^ (Table S1) were collected using both 850 MHz and 600 MHz spectrometers. Representative spectra from both brands were superimposed and visually appeared similar (Figure 2, left). Spectral regions belonging to excipients, residual solvents, water and blank were excluded before PCA. The PCA results showed that the first two principal components accounted for over 70% of the spectral intensity variations (Figure 2, right). The Mahalanobis distance (D_M_) calculated from the first three principal components (Table S2) using Equations (1) and (2) were 1.95 and 3.15, when the 850 MHz data and the 600 MHz data were used, respectively. Both values were below the established similarity threshold D_M_ value of 3.3 [27], suggesting similar HOS between the two products. Ninety percent (90%) confidence interval ellipses were drawn for the Rituxan^®^ DP lots in PC1/2 space (Figure 2, right). For the 850 MHz data, two lots of Reditux^®^ fell outside the ellipse; for the 600 MHz data, one lot of Reditux^®^ fell outside the ellipse. Therefore, the slight difference in field dependent D_M_ values is not necessarily correlated to the apparent differences shown in the ellipse circles. The results suggest any D_M_ values below the metric of 3.3 would indicate high similarity.

### 2.2. 2D ^1^H-^13^C Spectroscopy

#### 2.2.1. Protein Specificity

**Figure 3:**
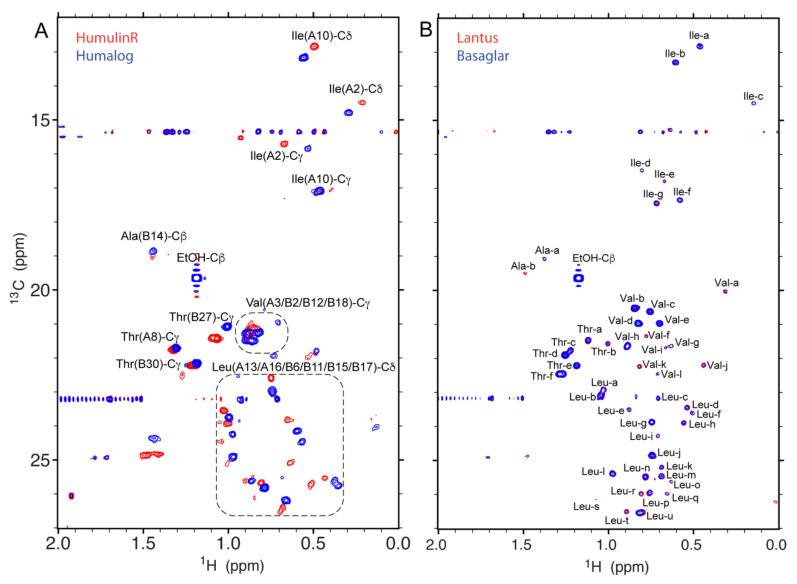
The superimposed 2D ^1^H-^13^C HSQC NMR spectra between insulin drug products of HumulinR^®^ and Humalog^®^ (**A**) and between Lantus^®^ and Basaglar^®^ (**B**) collected using a 600 MHz spectrometer. The plotting threshold of intensity was at a signal to noise ratio of 5 and 10 for (**A**) and (**B**), respectively. The methyl spectra of insulin human and insulin lispro were approximately assigned according to the published assignments; ambiguities were observed in the Valine and Leucine clusters shown in dashed boxes (**A**). The spectra of insulin glargine cannot be definitively assigned due to a large change in the observed chemical shifts and each peak was labeled for possible amino acid type and with an alphabetic letter (**B**).

As an alternative to 1D ^1^H spectra, 2D ^1^H-^13^C heteronuclear single quantum coherence (HSQC) spectra are highly specific to protein sequence and HOS. The HSQC spectrum of the methyl chemical shift region, which was 12–27 ppm along the ^13^C axis and 0–1.5 ppm along the ^1^H axis, has higher sensitivity due to the 3 C-H correlations and fast internal rotational dynamics of the methyl group. In addition, major excipient or solvent peaks, if observed in a methyl HSQC spectrum, can be readily identified because of their strong intensity and unique ^13^C chemical shifts, e.g., ethanol in insulin DPs of HumulinR^®^, Humalog^®^ and Basaglar^®^ (Figure 3). Therefore, methyl-HSQC spectra can be an ideal high-resolution spectrum for HOS assessment.

The amino acids possessing one or two methyl groups are Alanine (Ala), Methionine (Met), Threonine (Thr), Valine (Val), Leucine (Leu) and Isoleucine (Ile). For insulin, methyl peaks of Ala, Thr, Val, Leu and Ile observed in the DP spectra of HumulinR^®^ and Humalog^®^ (Table 1) can be approximately assigned by referencing the literature assignment for insulin human [39] and insulin lispro [40] (Figure 3A). Due to formulation or buffer differences, the assignment can be confidently made for residues of Ile at positions A2 and A10, Ala at position B14 and Thr at positions A8, B27 and B30; ambiguities remain for the Val and Leu clusters in the ^13^C chemical shift ranges of 21–22 ppm and 23–27 ppm, respectively (Figure 3A). Nevertheless, large chemical shift differences were observed between insulin human and insulin lispro, both of which have an identical M.W. of 5808 Da. The insulin lispro sequence differs from insulin human by two amino acid residues at positions B28–B29, Pro-Lys in insulin human and Lys-Pro in insulin lispro. Thus, the sequence difference introduced large changes in chemical shifts for almost every methyl peak (Figure 3A), which is consistent with a large change of HOS in the formulated API arising from only a two amino acids swap.

Shown in Figure 3B is the superimposed spectra between Lantus^®^ and Basaglar^®^, both of which contain the same DS insulin glargine. The chemical shift assignment was not transferrable from other insulins because the spectra are significantly different (Figure 3A,B). The methyl peaks of insulin glargine were labeled with possible amino acid type and alphabetic letters. The total number of identified peaks (*s*/*n* > 10) was 48, which is more than the expected 28 methyl peaks calculated from the insulin glargine sequence. The increased peak number is attributed to some of the methyl groups adopting at least two slow exchange conformations in the formulation, e.g., Ala(B14) had two peaks of Ala-a and Ala-b at ^13^C chemical shift of 19 ppm (Figure 3B). Overall, the methyl HSQC spectra between the two DPs are highly similar, suggesting that insulin glargine is folded in similar HOS for the two formulations.

#### 2.2.2. Similarity Metrics of Δδ

**Figure 4:**
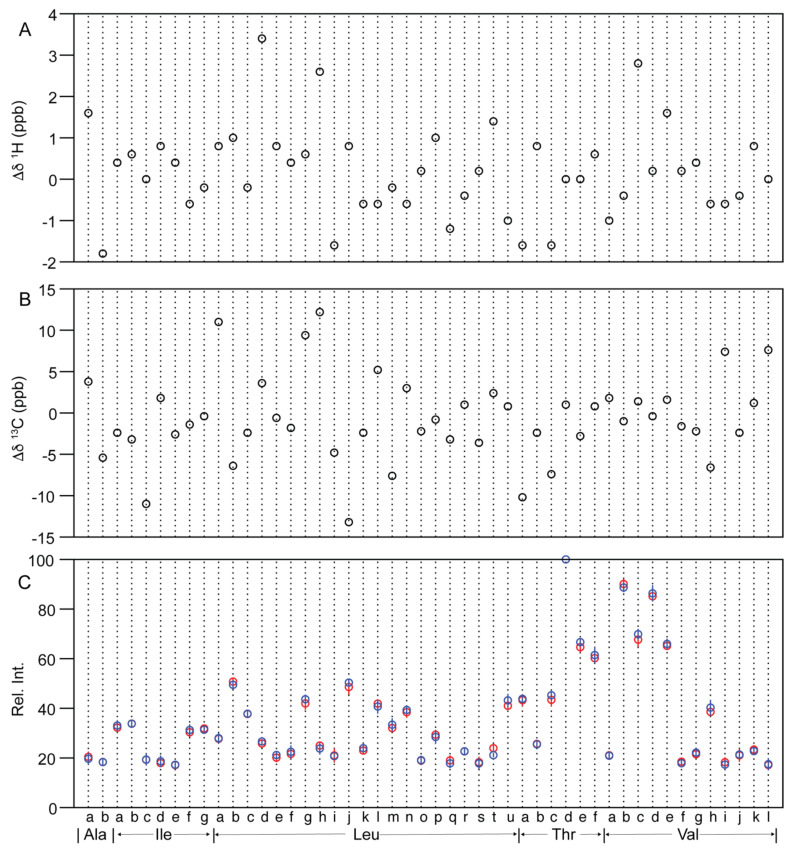
The chemical shift and relative peak height difference between insulin glargine drug products of Lantus^®^ and Basaglar^®^. The ^1^H (**A**) and ^13^C (**B**) chemical shift difference and the relative peak heights (**C**) were plotted along the labeled peaks of Figure 3B.

Each peak in a 2D ^1^H-^13^C NMR spectrum has three dimensions, including peak intensity and ^1^H and ^13^C chemical shifts, all of which are sensitive to protein HOS. Previous spectral comparisons on insulin [38] and filgrastim [26] 2D spectra have applied PCA for similarity evaluation, which took into account all spectral variables from the three dimensions (two frequencies and intensity) for comparison. However, no similarity metrics were derived. The filgrastim ^1^H-^15^N spectral comparison established a combined chemical shift difference (CCSD) metric of 8 ppb [26]. The chemical shift comparison was repeated here for the 48 methyl peaks between Lantus^®^ and Basaglar^®^. For each brand the inter-lot averaged chemical shift values were used as DP specific δ. The differences of chemical shift (Δδ) between the two DPs were plotted along both ^1^H and ^13^C axis (Figure 4A,B). The maximum ^1^H Δδ was 3.4 ppb identified in the Leu-d peak. The maximum ^13^C Δδ was −13 ppb identified in the Leu-j peak. When a 10% larger difference is permitted in the maximum Δδ, similarity metrics with rounded values of 4 and 15 ppb for the ^1^H and ^13^C chemical shifts, respectively, can be proposed. These metrics are on par with the previous CCSD metric of 8 ppb [26] or 4 ppb [41], which was a normalized Δδ value from both the ^1^H and ^15^N axes.

#### 2.2.3. Methyl Peak Profile

The peak intensity was compared using peak heights. First, the absolute peak heights of the strongest peak, Thr-d, were tabulated for five lots of each brand and five technical repeats from one lot of Lantus^®^ (Table S3). The calculated *p* value between the five technical repeats and the five lots of Lantus^®^ was 0.35, demonstrating the technical issues related to the spectral differences were within the inter-lot DP differences. By contrast, the Thr-d peak height in Lantus^®^ inter-lot spectra was on average 4% higher than the peak height of the Basaglar^®^ inter-lot spectra. The 4% difference was significant with a *p* value of 0.0061 (Table S3), which is less than the threshold value of 0.05. The 4% difference may be related to differences in assay and response *Q*-factor of the NMR probe to different formulations, usually related to electric capacity or ionic strength [42]. As a result, the comparison using absolute peak height for all methyl peaks was deemed not suitable. 

However, the relative peak heights, related to the dynamics and exchange kinetics of each methyl group should still be a valid choice for comparison purposes. Here, the ratios of each peak height to the Thr-d were calculated according to Equation (3). The mean and standard deviation from both Lantus^®^ inter-lot spectra and Basaglar^®^ inter-lot spectra were plotted in Figure 4C. The *p* values were calculated for all 48 peaks (Table 2) and 47 *p* values were higher than 0.05 except for the Leu-t peak with *p* value of 0.0055. Ultimately, 47 out of 48 peaks were equivalent in relative peak height between the two brands, demonstrating that the HOS distribution and exchange kinetics of the insulin glargine in the two DPs were similar. The work suggested the similarity metrics for peaks that show comparable relative peak height could be at least 98% (47/48).

### 2.3. 2D ^1^H-^15^N Spectroscopy

**Figure 5:**
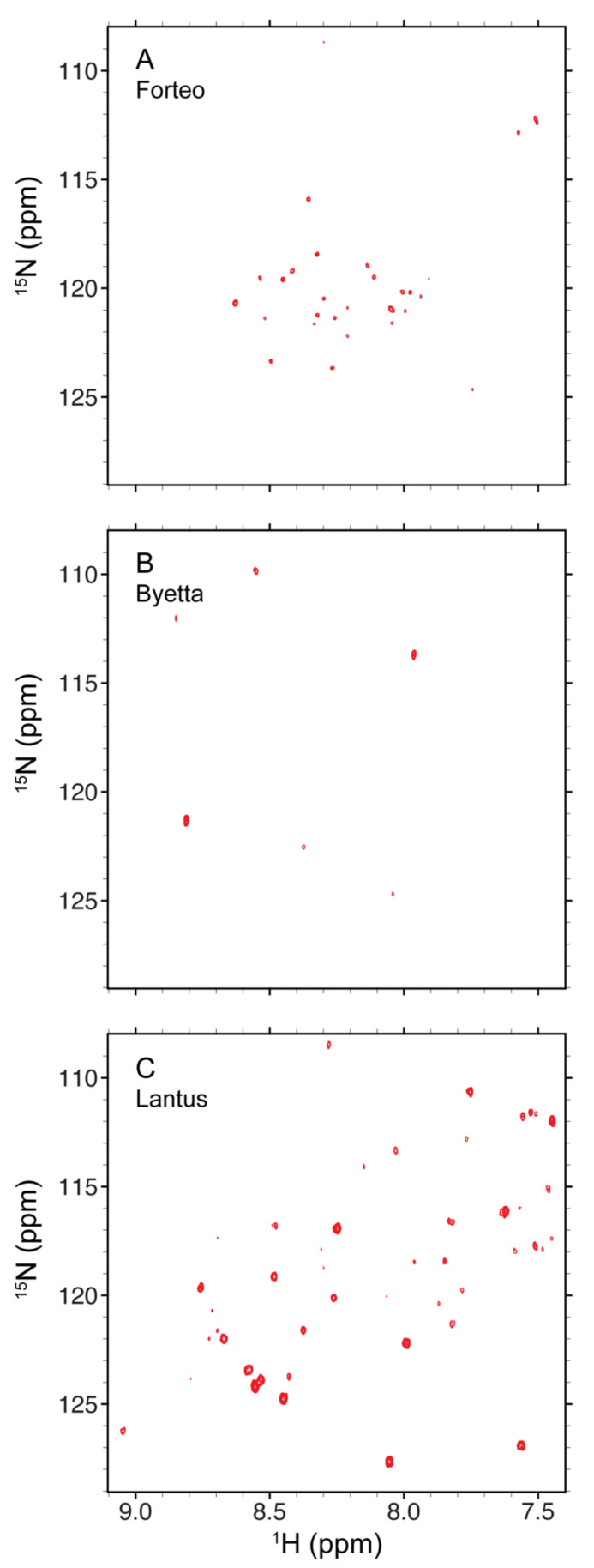
The 2D sofast ^1^H-^15^N HMQC NMR spectra of teriparatide in Forteo^®^ (**A**), exenatide in Byetta^®^ (**B**) and insulin glargine in Lantus^®^ (**C**) collected using an 850 MHz spectrometer. The plotting threshold of intensity was at a signal to noise ratio of 5.

The 2D ^1^H-^15^N spectrum may be a more specific NMR experiment than ^1^H-^13^C HSQC to evaluate protein HOS because the amide ^1^H and ^15^N chemical shifts are exclusively sensitive to peptide backbone conformation. However, the ^1^H-^15^N HSQC experiment is at least one order of magnitude less sensitive than the methyl ^1^H-^13^C HSQC experiment. Thus, ^15^N spectra via indirect detection in formulated DP samples can be challenging to collect when the DS concentration is less than 1 mM. The previously developed NMR pulse sequence of sofast-heteronuclear multi-quantum correlation (HMQC) has the advantage of shorter recycle delay without perturbing water resonances [43]. The sofast-HMQC experiment allows the 2D ^1^H-^15^N correlation spectrum to be collected within 24 h for DPs with protein concentrations as low as 0.06 mM. Representative spectra of Forteo^®^, Byetta^®^ and Lantus^®^ are shown in Figure 5. Amide peaks of the protein backbone and Asn/Gln side chains are observed in the ^15^N chemical shift range of 108–129 ppm and the ^1^H chemical shift range of 7.4–9.1 ppm. The number of detected peaks for teriparatide in Forteo^®^ was 29 (Figure 5A), while a total of 44 peaks are expected. The 66% coverage suggests the teriparatide adopts a well-defined HOS in the formulation, which is consistent with the 1D ^1^H spectral pattern (Figure 1C). By contrast, only six peaks were detected for exenatide in Byetta^®^ (Figure 5B), whereas a total of 39 peaks are expected. The 15% coverage suggests the exenatide resonances are in intermediate exchange between different HOS forms, which is, again, consistent with the broadening in the 1D ^1^H spectrum (Figure 1D). For insulin glargine in Lantus^®^, the 54 peaks detected account for 87% of the expected 62 peaks (Figure 5C). The results for insulin glargine suggest the existence of a single or fast averaged backbone HOS in the formulation.

## 3. Materials and Methods

### 3.1. Drug Product NMR Samples

All the drug products (DP) listed in Table 1 were sourced from the US market except Reditux^®^, which was sourced from the India market. The DPs used for similarity metrics calculations were 7 lots of Rituxan^®^, 3 lots of Reditux^®^, 5 lots of Lantus^®^ and 5 lots of Basaglar^®^ (Table S1). NMR samples were prepared by directly mixing 0.5 mL of DP formulation with 0.03 mL of deuterium oxide, which contained 0.002% of trimethylsilylpropanoic acid (TMSP) or trimethylsilylpropanesulfonate sodium (DSS), then transferring to a 5 mm NMR precision tube (Wilmad-LabGlass).

### 3.2. NMR Spectrsocopy

All the NMR spectra were collected at experimental temperature of 25 °C. The NMR spectrometers were either a Bruker (Billerica, MA, USA) 850 MHz equipped with a cryogenic QCI probe or a Bruker 600 MHz equipped with a liquid nitrogen-cooled prodigy TCI probe. 

#### 3.2.1. 1D ^1^H NMR Spectra Collection and Processing

The 1D ^1^H NMR spectra shown in Figure 1 and Figure 2A were collected using an 850 MHz spectrometer. The pulse program *p3919gp* was applied. The ^1^H carrier was placed on the water resonance at 4.8 ppm. The spectral width was 14 ppm and a total of 23,808 complex points were collected. The acquisition time was 1 s and recycle delay was 2 s. The number of scans were 1024 for calcitonin-salmon and rituximab DPs, 256 for exenatide DP and 128 for liraglutide, teriparatide and insulin glargine DPs. Each free induction decay (FID) was apodized with a 90° shifted sine-square window function, scaled half for the first point, zero-order phase corrected and zero filled to a spectral size of 32k points before Fourier transform (FT). A baseline correction method of splines was applied for the calcitonin-salmon and teriparatide spectra and no correction was applied for the liraglutide, exenatide, insulin glargine and rituximab spectra. All the 1D NMR data were processed and analyzed using MestReNova 14.1 software (Mestrelab Research S.L.).

The 1D ^1^H NMR spectra shown in Figure 2B were collected using a 600 MHz spectrometer. The pulse program of modified 1D NOESY *noe-p3919.kc* was applied [20]. The ^1^H carrier was placed on the water resonance at 4.8 ppm. The spectral width was 13 ppm and a total of 16,384 complex points were collected. The acquisition time was 1 s and the recycle delay was 2 s. The NOE mixing time was 0.1 s. The number of scans was 1024. The NMR samples and data processing were identical to that used for the 850 MHz spectra.

#### 3.2.2. 2D ^1^H-^13^C NMR Spectra Collection and Processing

The 2D ^1^H-^13^C HSQC spectra shown in Figure 3 were collected using a 600 MHz spectrometer. A modified sensitivity enhanced gradient HSQC pulse sequence *hsqcetgpsi2.kc* was applied [44]. The spectral width for the ^1^H dimension was 11 ppm with the carrier frequency centered at 4.8 ppm. The spectral width for the ^13^C dimension was 50 ppm with the carrier frequency centered at 23 ppm. The complex points of 1024 and 600 were acquired for the ^1^H and ^13^C dimensions, respectively. The resulting acquisition times for ^1^H and ^13^C spins were 78 and 40 ms, respectively. The ^13^C decoupling sequence was GARP with a radio frequency field strength of 1.9 kHz. The coupling constant ^1^J_HC_ was set to 155 Hz as a compromise between efficient INEPT transfer and T_2_ signal loss. The recycle delay was 2 s. The number of scans was 16 and the total experimental time was 6 h.

The data processing was performed using NMRPipe [45]. The apodization function of cosine was applied to both dimensions of ^1^H and ^13^C. The first point was scaled with a factor of 0.5 before zero-order phase correction. Zero filling of 2048 × 1024 real data points was applied to the ^1^H and ^13^C dimensions. The baseline corrections on frequency domains were carried out with a polynomial function under auto mode. The chemical shift reference followed the established procedure [46]. HSQC peaks with *s*/*n* higher than 10 were picked and peak heights were recorded using Sparky (Sparky 3, UCSF).

#### 3.2.3. 2D ^1^H-^15^N NMR Spectra Collection and Processing

The sofast 2D ^1^H-^15^N HMQC spectra shown in Figure 5 were collected using an 850 MHz spectrometer. The Bruker pulse sequence of *sfhmqcf3gpph* was applied. The spectral width for the ^1^H dimension was 14 ppm with the carrier frequency centered at 4.8 ppm. The spectral width for the ^15^N dimension was 35 ppm with the carrier frequency centered at 117 ppm. The complex points of 1784 and 200 were acquired for the ^1^H and ^15^N dimensions, respectively. The resulting acquisition times for ^1^H and ^15^N spins were 75 and 33 ms, respectively. The ^15^N decoupling sequence was GARP with a radio frequency field strength of 1.1 kHz. The coupling constant ^1^J_HN_ was set to 100 Hz as a compromise between efficient INEPT transfer and T_2_ signal loss. The recycle delay was 0.1 s. The number of scans was 2000 and the total experimental time was 23 h. The ^1^H-^15^N spectra were processed in a manner similar to the ^1^H-^13^C spectra, except for the zero filling of 4096 × 1024 real data points applied to the ^1^H and ^15^N dimensions. 

### 3.3. Calculation of Similarity Metrics

The similarity metrics were calculated using the above processed 1D ^1^H spectra of Rituxan^®^ and Reditux^®^ and 2D ^1^H-^13^C spectra of Lantus^®^ and Basaglar^®^. 

#### 3.3.1. Mahalanobis Distance (D_M_) between 1D Spectra

The 1D ^1^H NMR spectra of Rituxan^®^ and Reditux^®^ were used to calculate Mahalanobis distance [47]. The procedure was described previously [27]. Briefly, principal component analysis (PCA) was performed on the spectra of 7 lots of Rituxan^®^ and 3 lots of Reditux^®^. The spectral regions corresponding to peaks of excipient and solvent were excluded, including regions of 0–0.2 ppm, 0.85–0.95 ppm, 1.15–1.45 ppm, 1.55–1.65 ppm, 1.9–2.1 ppm, 2.2–2.9 ppm, 3.3–6 ppm and 8.45–8.47. The rest of the spectra were binned at 0.01 ppm resolution, resulting in a total of 370 bins with summed spectral intensities within each bin. The summed intensities were subject to integrity checks, sum normalization and Pareto scaling before PCA using MestReNova 14.1 (Mestrelab Research S.L.).

The Mahalanobis distances (*D_M_*) between the two rituximab DPs were calculated using PC1-3 scores. PCA scores from all the lots of each brand were tabulated as sample matrices of $A_{m\times p}$ and $B_{n\times p}$ for Rituxan^®^ and Reditux^®^, respectively, with m or n representing the number of lots and *p* representing the number of principal components used toward *D_M_* calculation. In the present study, m was 7 for Rituxan^®^, n was 3 for Reditux^®^ and *p* was 3. The mean vector ${\bar{A}}_{1\times p}$ and covariance matrix $S_{A,p\times p}$ was calculated using Rituxan^®^ sample matrix $A_{m\times p}$. In parallel, the mean vector ${\bar{B}}_{1\times p}$ and covariance matrix $S_{B,p\times p}$ was calculated using Reditux^®^ sample matrix $B_{n\times p}$. The covariance matrices of the two were averaged per Equation (1) before calculating *D_M_* using Equation (2). The calculations were performed using MATLAB 9.0 (The MathWorks Inc.) and the code can be found in the Supplementary Materials.

(1) $$S=\left(mS_{A}+nS_{B}\right)/\left(m+n\right)$$

(2) $$D_{M}=\sqrt{\left(\;\bar{A}-\bar{B}\right)S^{-1}\left(\bar{A}-\bar{B}\right)\prime }$$

#### 3.3.2. Chemical Shift Difference (Δδ) between 2D Spectra

The 2D ^1^H-^13^C NMR spectra of Lantus^®^ and Basaglar^®^ were used to calculate the chemical shift difference (Δδ). A total of 48 methyl peaks were identified with a signal to noise (*s*/*n*) ratio over 10. The peaks were approximately assigned to amino acid residue types of Ala, Thr, Ile, Leu and Val. Within each amino acid residue type, the peaks were labeled with alphabetic letters. The ^1^H and ^13^C chemical shift of each brand were averaged from the spectra of the 5 lots, representing the mean chemical shift of the peak in each brand. Chemical shift difference (Δδ) was the difference between the mean values of Basaglar^®^ and Lantus^®^.

#### 3.3.3. Methyl Peak Profile between 2D Spectra

The peak heights of the 48 identified methyl peaks in 2D ^1^H-^13^C spectra of Lantus^®^ and Basaglar^®^ were recorded as *I_x_* for peak *x*. The relative peak intensity of the peak *x* (*Rel.Int._x_*) was calculated per Equation (3), where *I_Thr-d_* is the peak height of the peak Thr-d, which is the peak with the highest intensity. The mean and standard deviation of *Rel.Int._x_* were calculated from the spectra of 5 different lots within each brand (Table 2). The *p* value was calculated for each peak *x* using *t*-test function of two-sample assuming unequal variances in Excel (ver. 16.46). The significant threshold of 0.05 in *p* value was used to determine the equivalence of relative peak heights. The equation for relative peak intensity is described as follows.

(3) $$Rel.Int._{x}=100\times I_{x}/I_{Thr-d}$$

## 4. Discussion and Conclusions

### 4.1. HOS Inferrred from 1D and 2D Spectra

In this work, standard NMR experiments using 1D ^1^H, 2D ^1^H-^13^C HSQC and 2D ^1^H-^15^N sofast HMQC pulse sequences were performed on formulated protein DPs. The NMR peak patterns from both 1D and 2D spectra are qualitatively informative for protein HOS properties, providing insight into the oligomerization of liraglutide, the HOS exchange of exenatide and the well folded HOS of calcitonin-salmon, teriparatide, insulin glargine and rituximab. In general, the 1D ^1^H NMR experiment provides information on the HOS profile and whether a protein is folded in formulation. Information on more specific HOS variation can be obtained from heteronuclear 2D spectra. Each 2D spectrum was sensitive to different aspect of HOS. For example, in the insulin glargine spectra, while the methyl ^1^H-^13^C spectrum showed the sidechains adopting two slowly exchanging conformers, the ^1^H-^15^N spectrum was more consistent with a single well-folded backbone conformer. The two observations were not necessarily inconsistent with each other, rather, they illustrate the complex nature of protein HOS in the formulation and the atomic level probes used by the different NMR experiments.

### 4.2. HOS Similarity Metrics Calculated from 1D and 2D Spectra

What is different from the pioneering work on demonstrating heteronuclear 2D NMR at protein natural abundance [41,48,49] is that the current study uses NMR on formulated DPs and also includes deriving practically achievable similarity metrics. Earlier work demonstrated the practically achievable Mahalanobis distance (D_M_) value of 3.3 based on the PCA of 1D ^1^H spectra collected on the marketed insulin reference product and follow-on products [27]. Here, we obtained the D_M_ values of 1.95 and 3.15 using PCA and 1D ^1^H spectra of rituximab DPs marketed in the US and India, suggesting that a D_M_ metrics value of less than 3.3 could be a general acceptance criterion.

While PCA can be conveniently performed on 1D spectra and has been demonstrated on 2D spectra [26,38,50], PCA is challenging to implement for 2D spectra because of the technical complications in binning the 2D spectra and avoiding non-DS peaks at the same time. An alternative method is to focus on the DS peak profile. The normalized distance comparison approach was proposed to compare 2D spectra along the axes of chemical shifts and peak intensity; however, no acceptance criteria were ever proposed [51]. Here the previous chemical shift comparison method [26] was verified using 2D ^1^H-^13^C spectra collected on insulin glargine DPs and the chemical shift different metrics (Δδ) of 4 ppb for ^1^H and 15 ppb for ^13^C were derived. Furthermore, the peak profile method [44] was adopted to compare the relative peak heights between two insulin glargine brands, where *p* values were derived from *t*-test. In these insulin spectra, 98% of the methyl cross peaks had equivalent relative peak heights between the two brands. These 2D spectral similarity metrics could be equivalent to the D_M_ value of 1.6 obtained by using 1D spectra [27]. The methyl peak profile results represent another practically achievable similarity metrics for 2D spectral comparison.

In summary, the NMR data collected in the current study provided examples of simple experiments and analyses on formulated protein DP and demonstrated practical measurements to assess equivalence of HOS between different DPs. The metrics proposed were validated using marketed similar DPs that were manufactured differently and are proposed as a benchmark to determine the degree of similarity for protein HOS in formulated DPs.

## Figures and Tables

**Table 1:** Drug Products studied.

Drug Product	Drug Substance	Number of Amino Acids	M.W. (kDa)	Concentration (mM)	pH
Miacalcin^®^	Calcitonin-Salmon	32	3.43	0.0097 ^1^	n/a
Saxenda^®^	Liraglutide	38	3.75	1.6	8.15
Forteo^®^	Teriparatide	34	4.12	0.061	4
Byetta^®^	Exenatide	39	4.19	0.060	4.5
HumulinR^®^	Insulin Human	51	5.81	0.60 ^2^	n/a
Humalog^®^	Insulin Lispro	51	5.81	0.60 ^2^	7.0–7.8
Lantus^®^Basaglar^®^	Insulin Glargine	53	6.06	0.60	4
Rituxan^®^Reditux^®^	Rituximab	1328	145	0.069	6.5

^1^ Based on the equivalence between 1 mg and 6000 I.U. per USP NF.; ^2^ based on the equivalence between 0.0347 mg and 1 USP unit per USP NF and Eu. Pharm.; n/a: not available from the drug label.

**Table 2:** The relative peak height comparison of insulin glargine DPs.

Peak	Antus^®^					Basaglar^®^					*p* Value
	Lot 1	Lot 2	Lot 3	Lot 4	Lot 5	Lot 1	Lot 2	Lot 3	Lot 4	Lot 5	
Ala-a	17.5	20.7	19.8	20.8	23.4	20.2	17.0	22.7	20.8	17.6	0.61
Ala-b	18.0	19.0	17.4	18.1	19.5	16.3	18.8	17.3	18.7	20.7	0.96
Ile-a	30.2	34.7	32.7	30.6	32.8	34.5	33.2	29.6	35.5	32.4	0.54
Ile-b	35.3	34.5	33.6	32.5	33.3	34.9	34.3	32.2	34.1	33.3	0.92
Ile-c	20.1	21.9	19.2	17.8	17.4	20.9	21.1	15.5	18.3	21.0	0.96
Ile-d	20.0	16.8	17.8	16.9	18.0	19.8	20.8	17.2	15.2	20.2	0.57
Ile-e	16.9	15.9	16.4	17.3	19.2	19.3	15.7	16.5	16.7	17.9	0.93
Ile-f	29.8	32.3	27.4	30.4	31.9	30.6	31.4	35.0	28.7	30.7	0.52
Ile-g	32.0	31.4	30.6	30.9	34.6	31.0	32.6	31.8	31.0	30.4	0.54
Leu-a	27.6	26.4	26.7	27.2	30.7	29.1	27.9	25.4	26.6	30.8	0.84
Leu-b	52.8	52.0	48.8	49.4	50.7	50.9	49.3	50.3	46.5	50.3	0.28
Leu-c	36.9	37.6	36.3	39.3	38.4	39.9	35.9	38.0	38.3	37.2	0.88
Leu-d	25.7	25.9	26.7	24.8	25.8	26.6	25.3	27.0	27.0	27.2	0.11
Leu-e	22.0	17.7	18.8	20.9	21.1	23.4	20.9	22.3	19.4	20.0	0.35
Leu-f	22.5	18.8	21.6	21.2	24.0	23.6	25.7	22.1	18.5	22.3	0.58
Leu-g	41.1	41.1	39.6	41.7	46.2	44.7	43.6	43.5	40.8	45.7	0.26
Leu-h	23.3	26.6	23.6	25.6	25.8	25.9	24.6	21.2	21.6	25.7	0.35
Leu-i	20.1	22.2	16.9	21.1	24.8	21.3	21.0	21.1	20.2	19.9	0.83
Leu-j	46.4	50.1	44.5	49.9	52.0	51.8	50.8	48.3	49.6	51.1	0.28
Leu-k	21.7	25.4	21.7	23.1	23.0	26.2	22.1	23.7	24.9	22.6	0.38
Leu-l	42.4	41.8	41.6	41.3	42.3	43.9	41.4	37.5	38.0	42.9	0.43
Leu-m	31.2	31.0	32.7	31.2	33.9	34.2	34.6	29.1	32.9	36.0	0.34
Leu-n	36.2	38.2	37.2	38.4	42.3	40.8	37.6	37.3	40.5	40.5	0.52
Leu-o	18.1	17.6	18.8	21.4	18.5	18.8	17.3	20.2	21.6	17.7	0.85
Leu-p	30.6	30.6	29.4	28.0	28.1	31.7	28.7	24.7	26.4	30.8	0.57
Leu-q	19.1	21.8	19.7	16.1	18.3	18.6	19.9	15.1	16.7	18.7	0.38
Leu-r	24.2	22.7	23.0	21.9	21.5	22.6	24.7	23.5	20.5	22.2	0.96
Leu-s	15.3	19.1	18.4	17.3	21.4	15.7	17.9	15.6	18.9	21.0	0.75
Leu-t	23.0	25.3	23.7	25.3	22.4	21.7	21.5	21.6	19.6	21.3	0.0055
Leu-u	42.5	38.8	40.1	41.6	42.2	43.2	40.2	42.2	42.8	47.5	0.17
Thr-a	45.8	45.8	41.2	40.9	42.8	43.8	41.9	44.8	42.4	46.0	0.73
Thr-b	27.2	27.0	24.0	25.0	25.0	24.1	25.8	26.1	25.9	25.5	0.83
Thr-c	39.4	45.2	42.5	43.8	46.1	45.9	44.2	47.3	44.7	44.3	0.21
Thr-d	100	100	100	100	100	100	100	100	100	100	n/a
Thr-e	64.0	65.5	61.5	64.8	67.4	68.5	68.0	64.5	65.5	66.9	0.13
Thr-f	60.2	61.1	59.0	58.8	61.9	65.1	64.5	58.8	58.1	60.7	0.46
Val-a	22.5	21.6	22.1	19.1	20.0	22.3	21.7	18.7	20.6	21.3	0.85
Val-b	92.1	91.1	87.1	88.6	91.4	91.2	86.6	87.4	88.4	89.6	0.29
Val-c	67.2	65.7	64.6	68.9	71.8	73.0	70.7	67.1	69.2	69.6	0.2
Val-d	84.8	84.8	82.9	87.1	86.3	89.3	90.4	83.1	85.1	83.9	0.5
Val-e	64.2	65.2	63.6	67.1	65.6	68.1	67.2	62.5	66.4	65.3	0.52
Val-f	20.0	17.8	19.3	18.0	17.4	18.4	17.2	17.3	19.2	17.2	0.34
Val-g	20.9	20.2	21.8	21.3	23.5	20.9	21.6	21.1	22.1	25.1	0.52
Val-h	40.0	37.7	37.8	37.6	39.6	43.3	36.4	40.0	38.3	43.3	0.29
Val-i	15.9	20.7	17.5	17.3	20.0	18.1	19.3	16.4	15.4	17.4	0.42
Val-j	24.0	20.8	21.9	16.8	21.7	22.9	23.2	20.9	20.3	19.4	0.83
Val-k	24.2	21.1	25.7	22.8	23.0	21.5	21.9	24.0	24.3	22.0	0.55
Val-l	15.8	19.9	14.5	17.9	17.7	20.2	13.5	17.1	18.6	18.0	0.84

n/a: not available.

## Data Availability

The data presented in this study are available in supplementary material.

## Supplementary Materials

The following are available online. Tables S1–S3 and Matlab code for D_M_ calculation.

## Sample Availability

Samples of the compounds are not available from the authors.

## References

[1] Zhang L., Lionberger R.A. (2020). Generics 2030: Where Are We Heading in 2030 for Generic Drug Science, Research, and Regulation?. Clin. Pharmacol. Ther..

[2] Christl L.A., Woodcock J., Kozlowski S. (2017). Biosimilars: The US Regulatory Framework. Annu. Rev. Med..

[3] Fisher A.C., Lee S.L., Harris D.P., Buhse L., Kozlowski S., Yu L., Kopcha M., Woodcock J. (2016). Advancing pharmaceutical quality: An overview of science and research in the US FDA’s Office of Pharmaceutical Quality. Int. J. Pharm..

[4] Kozlowski S., Woodcock J., Midthun K., Behrman Sherman R. (2011). Developing the Nation’s Biosimilars Program. N. Engl. J. Med..

[5] Moussa E.M., Panchal J.P., Moorthy B.S., Blum J.S., Joubert M.K., Narhi L.O., Topp E.M. (2016). Immunogenicity of Therapeutic Protein Aggregates. J. Pharm. Sci..

[6] Gruia F., Du J., Santacroce P.V., Remmele R.L., Bee J.S. (2015). Technical Decision Making with Higher Order Structure Data: Impact of a Formulation Change on the Higher Order Structure and Stability of a mAb. J. Pharm. Sci..

[7] Aubin Y., Hodgson D.J., Thach W.B., Gingras G., Sauvé S. (2015). Monitoring Effects of Excipients, Formulation Parameters and Mutations on the High Order Structure of Filgrastim by NMR. Pharm. Res..

[8] Bramham J.E., Podmore A., Davies S.A., Golovanov A.P. (2020). Comprehensive Assessment of Protein and Excipient Stability in Biopharmaceutical Formulations Using 1H NMR Spectroscopy. Acs Pharmacol. Transl. Sci..

[9] Panjwani N., Hodgson D.J., Sauvé S., Aubin Y. (2010). Assessment of the Effects of pH, Formulation and Deformulation on the Conformation of Interferon Alpha-2 by NMR. J. Pharm. Sci..

[10] Shah D.D., Singh S.M., Mallela K.M.G. (2018). Effect of Chemical Oxidation on the Higher Order Structure, Stability, Aggregation, and Biological Function of Interferon Alpha-2a: Role of Local Structural Changes Detected by 2D NMR. Pharm. Res..

[11] Hodgson D.J., Aubin Y. (2017). Assessment of the structure of pegylated-recombinant protein therapeutics by the NMR fingerprint assay. J. Pharm. Biomed. Anal..

[12] Casagrande F., Dégardin K., Ross A. (2020). Protein NMR of biologicals: Analytical support for development and marketed products. J. Biomol. NMR.

[13] Weiss W.F., Gabrielson J.P., Al-Azzam W., Chen G., Davis D.L., Das T.K., Hayes D.B., Houde D., Singh S.K. (2016). Technical Decision Making with Higher Order Structure Data: Perspectives on Higher Order Structure Characterization from the Biopharmaceutical Industry. J. Pharm. Sci..

[14] Keire D.A., Sasisekharan R., Lee S.L., Rosenberg A., Walker L.A. (2019). Analytical Tools for Physicochemical Characterization and Fingerprinting. Science and Regulations of Naturally Derived Complex Drugs.

[15] Guerrini M., Rudd T.R., Yates E.A., Sasisekharan R., Lee S.L., Rosenberg A., Walker L.A. (2019). NMR in the Characterization of Complex Mixture Drugs. Science and Regulations of Naturally Derived Complex Drugs.

[16] Amezcua C.A., Szabo C.M. (2013). Assessment of Higher Order Structure Comparability in Therapeutic Proteins Using Nuclear Magnetic Resonance Spectroscopy. J. Pharm. Sci..

[17] Arbogast L.W., Brinson R.G., Formolo T., Hoopes J.T., Marino J.P. (2016). 2D 1HN, 15N Correlated NMR Methods at Natural Abundance for Obtaining Structural Maps and Statistical Comparability of Monoclonal Antibodies. Pharm. Res..

[18] Arbogast L.W., Brinson R.G., Marino J.P. (2015). Mapping Monoclonal Antibody Structure by 2D 13C NMR at Natural Abundance. Anal. Chem..

[19] Kiss R., Fizil Á., Szántay C. (2018). What NMR can do in the biopharmaceutical industry. J. Pharm. Biomed. Anal..

[20] Chen K., Long D.S., Lute S.C., Levy M.J., Brorson K.A., Keire D.A. (2016). Simple NMR methods for evaluating higher order structures of monoclonal antibody therapeutics with quinary structure. J. Pharm. Biomed. Anal..

[21] Hodgson D.J., Ghasriani H., Aubin Y. (2019). Assessment of the higher order structure of Humira^®^, Remicade^®^, Avastin^®^, Rituxan^®^, Herceptin^®^, and Enbrel^®^ by 2D-NMR fingerprinting. J. Pharm. Biomed. Anal..

[22] Japelj B., Ilc G., Marušič J., Senčar J., Kuzman D., Plavec J. (2016). Biosimilar structural comparability assessment by NMR: From small proteins to monoclonal antibodies. Sci. Rep..

[23] Elliott K.W., Ghasriani H., Wikström M., Giddens J.P., Aubin Y., Delaglio F., Marino J.P., Arbogast L.W. (2020). Comparative Analysis of One-Dimensional Protein Fingerprint by Line Shape Enhancement and Two-Dimensional 1H,13C Methyl NMR Methods for Characterization of the Higher Order Structure of IgG1 Monoclonal Antibodies. Anal. Chem..

[24] Franks J., Glushka J.N., Jones M.T., Live D.H., Zou Q., Prestegard J.H. (2016). Spin Diffusion Editing for Structural Fingerprints of Therapeutic Antibodies. Anal. Chem..

[25] Poppe L., Jordan J.B., Lawson K., Jerums M., Apostol I., Schnier P.D. (2013). Profiling Formulated Monoclonal Antibodies by 1H NMR Spectroscopy. Anal. Chem..

[26] Ghasriani H., Hodgson D.J., Brinson R.G., McEwen I., Buhse L.F., Kozlowski S., Marino J.P., Aubin Y., Keire D.A. (2016). Precision and robustness of 2D-NMR for structure assessment of filgrastim biosimilars. Nat. Biotechnol..

[27] Wang D., Park J., Patil S.M., Smith C.J., Leazer J.L., Keire D.A., Chen K. (2020). An NMR-Based Similarity Metric for Higher Order Structure Quality Assessment Among, U.S. Marketed Insulin Therapeutics. J. Pharm. Sci..

[28] Ionova Y., Wilson L. (2020). Biologic excipients: Importance of clinical awareness of inactive ingredients. PLoS ONE.

[29] Wishart D.S., Knox C., Guo A.C., Eisner R., Young N., Gautam B., Hau D.D., Psychogios N., Dong E., Bouatra S. (2009). HMDB: A knowledgebase for the human metabolome. Nucleic Acids Res..

[30] Ulrich E.L., Akutsu H., Doreleijers J.F., Harano Y., Ioannidis Y.E., Lin J., Livny M., Mading S., Maziuk D., Miller Z. (2008). BioMagResBank. Nucleic Acids Res..

[31] Patil S.M., Li V., Peng J., Kozak D., Xu J., Cai B., Keire D.A., Chen K. (2019). A Simple and Noninvasive DOSY NMR Method for Droplet Size Measurement of Intact Oil-In-Water Emulsion Drug Products. J. Pharm. Sci..

[32] Wang W., Ignatius A.A., Thakkar S.V. (2014). Impact of Residual Impurities and Contaminants on Protein Stability. J. Pharm. Sci..

[33] Gottlieb H.E., Kotlyar V., Nudelman A. (1997). NMR Chemical Shifts of Common Laboratory Solvents as Trace Impurities. J. Org. Chem..

[34] Malmstrøm J. (2019). Quantification of Silicone Oil and Its Degradation Products in Aqueous Pharmaceutical Formulations by 1H-NMR Spectroscopy. J. Pharm. Sci..

[35] Suh M.S., Patil S.M., Kozak D., Pang E., Choi S., Jiang X., Rodriguez J.D., Keire D.A., Chen K. (2020). An NMR Protocol for In Vitro Paclitaxel Release from an Albumin-Bound Nanoparticle Formulation. AAPS Pharmscitech.

[36] Skidmore K., Hewitt D., Kao Y.-H. (2012). Quantitation and characterization of process impurities and extractables in protein-containing solutions using proton NMR as a general tool. Biotechnol. Prog..

[37] Palmer A.G., Kroenke C.D., Patrick Loria J., James T.L., Dötsch V., Schmitz U. (2001). Nuclear Magnetic Resonance Methods for Quantifying Microsecond-to-Millisecond Motions in Biological Macromolecules. Methods in Enzymology.

[38] Chen K., Park J., Li F., Patil S.M., Keire D.A. (2018). Chemometric Methods to Quantify 1D and 2D NMR Spectral Differences among Similar Protein Therapeutics. AAPS Pharmscitech.

[39] Chang X., Jørgensen A.M.M., Bardrum P., Led J.J. (1997). Solution Structures of the R6 Human Insulin Hexamer. Biochemistry.

[40] Quinternet M., Starck J.P., Delsuc M.A., Kieffer B. (2013). Heteronuclear NMR provides an accurate assessment of therapeutic insulin’s quality. J. Pharm. Biomed. Anal..

[41] Brinson R.G., Marino J.P., Delaglio F., Arbogast L.W., Evans R.M., Kearsley A., Gingras G., Ghasriani H., Aubin Y., Pierens G.K. (2019). Enabling adoption of 2D-NMR for the higher order structure assessment of monoclonal antibody therapeutics. mAbs.

[42] Nishizaki Y., Lankin D.C., Chen S.-N., Pauli G.F. (2021). Accurate and Precise External Calibration Enhances the Versatility of Quantitative NMR (qNMR). Anal. Chem..

[43] Schanda P., Brutscher B. (2005). Very Fast Two-Dimensional NMR Spectroscopy for Real-Time Investigation of Dynamic Events in Proteins on the Time Scale of Seconds. J. Am. Chem. Soc..

[44] Zhuo Y., Keire D.A., Chen K. (2021). Minor N-Glycan Mapping of Monoclonal Antibody Therapeutics Using Middle-Down NMR Spectroscopy. Mol. Pharm..

[45] Delaglio F., Grzesiek S., Vuister G.W., Zhu G., Pfeifer J., Bax A. (1995). Nmrpipe-a Multidimensional Spectral Processing System Based on Unix Pipes. J. Biomol. NMR.

[46] Wishart D.S., Bigam C.G., Yao J., Abildgaard F., Dyson H.J., Oldfield E., Markley J.L., Sykes B.D. (1995). 1H, 13C and 15N chemical shift referencing in biomolecular NMR. J. Biomol. NMR.

[47] Brereton R.G. (2015). The Mahalanobis distance and its relationship to principal component scores. J. Chemom..

[48] Aubin Y., Gingras G., Sauvé S. (2008). Assessment of the Three-Dimensional Structure of Recombinant Protein Therapeutics by NMR Fingerprinting:  Demonstration on Recombinant Human Granulocyte Macrophage-Colony Stimulation Factor. Anal. Chem..

[49] Chen K., Freedberg D.I., Keire D.A. (2015). NMR profiling of biomolecules at natural abundance using 2D 1H–15N and 1H–13C multiplicity-separated (MS) HSQC spectra. J. Magn. Reson..

[50] Arbogast L.W., Delaglio F., Schiel J.E., Marino J.P. (2017). Multivariate Analysis of Two-Dimensional 1H, 13C Methyl NMR Spectra of Monoclonal Antibody Therapeutics To Facilitate Assessment of Higher Order Structure. Anal. Chem..

[51] Haxholm G.W., Petersen B.O., Malmstrøm J. (2019). Higher-Order Structure Characterization of Pharmaceutical Proteins by 2D Nuclear Magnetic Resonance Methyl Fingerprinting. J. Pharm. Sci..

