# Supplementary material for: NMR Spectroscopy for Protein Higher Order Structure Similarity Assessment in Formulated Drug Products
Source: Molecules. 2021 Jul 13;26(14):4251. doi: 10.3390/molecules26144251 (PMC8307401; doi:10.3390/molecules26144251)
Supplement: Supplementary file 1 [file molecules-26-04251-s001.zip › molecules-1249044-supplementary.pdf]

NMR Spectroscopy for Protein Higher Order Structure Similarity Assessment in Formulated Drug Products

Deyun Wang [1], You Zhuo [2], Mike Karfunkle [3], Sharadrao M. Patil [2], Cameron J. Smith [4], David A. Keire [5] and Kang Chen [2,*]

Northeast Medical Products Laboratory, Office of Regulatory Science, Office of Regulatory Affairs, U.S. Food and Drug Administration, Jamaica, NY 11433, USA; deyun.wang@fda.hhs.gov

2 Division of Complex Drug Analysis, Office of Testing and Research, Office of Pharmaceutical Quality, Center for Drug Evaluation and Research, U.S. Food and Drug Administration, Silver Spring, MD 20993, USA; you.zhuo@fda.hhs.gov (Y.Z.); sharadmpatil@gmail.com(S.M.P.)

3 Division of Pharmaceutical Analysis, Office of Testing and Research, Office of Pharmaceutical Quality, Center for Drug Evaluation and Research, U.S. Food and Drug Administration, St. Louis, MO 63110, USA; mike.karfunkle@fda.hhs.gov

4 Division of Liquid Based Products I, Office of Lifecycle Drug Products, Office of Pharmaceutical Quality, Center for Drug Evaluation and Research, U.S. Food and Drug Administration, Silver Spring, MD 20993, USA; cameron.smith@fda.hhs.gov

5 Office of Testing and Research, Office of Pharmaceutical Quality, Center for Drug Evaluation and Research, U.S. Food and Drug Administration, St. Louis, MO 63110, USA; david.keire@fda.hhs.gov

* Correspondence: kang.chen@fda.hhs.gov; Tel.: 240-402-5550

Table S1. The DPs used for the similarity calculations.

DS	DP	Lot #	Expiry date
Rituximab	Rituxan®	626409	7/2016
		919860	5/2013
		3006206	7/2017
		3196990	1/2020
		3209283	3/2020
		3209284	5/2020
		3224628	5/2020
	Reditux®	RIAV01815	11/2017
		RIBV02412	5/2014
		RIBV03412	8/2014
Insulin glargine	Lantus®	8F5093A	06/2020
		8F5328A	09/2020
		8F5508A	02/2021
		9F5977A	09/2021
		9F6290A	02/2022
	Basaglar®	D011965C	12/2020
		C994684C	09/2020
		C882965A	12/2019
		C854573C	10/2019
		C837772C	09/2019

Table S2. The PC scores obtained for rituximab DPs.

DP	Lot #	850 MHz			600 MHz		
		PC1	PC2	PC3	PC1	PC2	PC3
Rituxan®	626409	0.119	-0.078	0.104	-0.080	0.122	-0.096
	919860	0.076	0.015	0.01	-0.227	-0.003	0.055
	3006206	0.003	-0.145	-0.070	-0.054	0.033	0.018
	3196990	-0.041	-0.017	-0.050	-0.046	-0.011	-0.028
	3209283	0.050	0.056	-0.024	0.016	-0.100	0.054
	3209284	0.044	0.064	-0.047	-0.092	-0.061	0.000
	3224628	0.024	0.009	0.007	0.042	-0.059	-0.031
Reditux®	RIAV01815	0.116	0.067	0.006	0.136	-0.085	-0.059
	RIBV02412	-0.187	0.023	0.027	0.131	0.194	0.054
	RIBV03412	-0.204	0.006	0.036	0.174	-0.03	0.034

Table S3. The absolute peak heights of the peak Thr-d from the spectra of Insulin glargine DPs.

<table>
<tr><th>Spectrum No.</th><th>Lantus® technical repeat</th><th>Lantus® inter-lot</th><th>Basaglar® inter-lot</th></tr>
<tr><td>1</td><td>4.48e6</td><td>4.39e6</td><td>4.08e6</td></tr>
<tr><td>2</td><td>4.45e6</td><td>4.25e6</td><td>4.15e6</td></tr>
<tr><td>3</td><td>4.39e6</td><td>4.47e6</td><td>4.24e6</td></tr>
<tr><td>4</td><td>4.35e6</td><td>4.38e6</td><td>4.26e6</td></tr>
<tr><td>5</td><td>4.36e6</td><td>4.32e6</td><td>4.16e6</td></tr>
<tr><td rowspan="2">p value</td><td colspan="2">0.35</td><td></td></tr>
<tr><td></td><td colspan="2">0.0061</td></tr>
</table>

Matlab code for D_M calculation

```
clear;
load test.list
load rld.list
A=rld;
B=test;
a=length(A);
b=length(B);
m=  mean(A)-mean(B);
covD=  (a*cov(A)+b*cov(B))/(a+b);
DM=  sqrt(m*inv(covD)*m')
```
